# Routine Use of a Standardized Mastectomy Diagram by Surgeons Improves Accuracy and Timeliness of the Final Pathological Report

**DOI:** 10.1245/s10434-023-14179-8

**Published:** 2023-08-19

**Authors:** Andrew Seto, Alexandra Pass, Robert Babkowski, Elgida R. Volpicelli, Zandra Cheng, Helen A. Pass

**Affiliations:** 1 Department of Surgery, Stamford Hospital, Stamford, CT USA; 2 Columbia University Vagelos College of Physicians and Surgeons, New York, NY USA; 3 New York University Grossman School of Medicine, New York, NY USA; 4 Department of Pathology, Stamford Hospital, Stamford, CT USA

**Keywords:** Breast cancer, Mastectomy, Diagram, Pathologic processing, Grossing

## Abstract

**Background:**

Accurate and timely assessment of pathology specimens is critical for patient care and oncologic management. This study aimed to determine whether a standardized mastectomy diagram would facilitate communication among surgeons and pathologists and improve pathologic processing.

**Methods:**

A prospective quality improvement study was conducted over a continuous 12-month period. During the first 6 months, usual pathologic processing of mastectomy specimens was performed per standard department protocol. In the second 6 months, a standardized mastectomy diagram was completed at the time of surgery, noting the location and preoperative pathologic diagnosis of all benign and malignant lesions. An analysis of covariance was used to compare the number of breast lesions identified and the number of days between specimen receipt and the date of the final pathology report between each group.

**Results:**

Time from specimen receipt to final pathologic report decreased from a mean (± SE) of 8.3 ± 0.7 days in the usual processing group to 6.1 ± 0.6 days with the use of the standardized mastectomy diagram, for a between-group difference of 2.1 days (95% confidence interval [CI] 0.3–4.0; *p* = 0.02). The number of lesions identified increased from 1.8 ± 0.2 to 2.6 ± 0.2, for a between-group difference of 0.8 (95% CI 0.1–1.5; *p* = 0.02).

**Conclusion:**

A standardized mastectomy diagram completed at the time of surgery improves the quality of pathologic processing. The diagram, which serves as a mastectomy lesion map, assists lesion localization, enhances accuracy, and reduces time to final pathology report.

Accurate and timely assessment of pathology specimens in breast carcinoma is critical for patient care and oncologic management. With the advances in treatment strategies and our understanding of breast cancer over the past few decades, the pathologic processing of mastectomy specimens has become progressively more challenging. Several factors contribute to the growing difficulty of pathologic processing of mastectomy specimens. First, there is a rising number of patients with early stage breast cancer and minimal disease burden that are choosing mastectomy for definitive treatment, resulting in an increase in mastectomy rates nationwide.^1,2^ This trend is seen across all age groups and in breast conserving surgery eligible patients.^3,4^ Larger specimens are inherently more difficult to process and lead to longer time from excision to final pathology report.

In addition, tissue processing is becoming more complex with the increased utilization of preoperative breast MRI. As the most sensitive imaging modality, preoperative MRI has led to the identification of additional foci of unsuspected malignant and benign lesions.^5,6^ It is also associated with significantly higher rates of mastectomy.^7,8^

Neoadjuvant chemotherapy increases the complexity of pathologic processing of mastectomy specimens as well. The tumor bed decreases in consistency and its extent becomes poorly defined.^9–11^ Residual tumor is harder to identify grossly, requiring thorough mapping and more extensive sampling. The response to neoadjuvant therapy along with the various histological variables that inform long-term prognosis has to be assessed. The phenotype may also need to be re-evaluated as neoadjuvant therapy can often change the hormone receptor and HER2 status.^12^ Compared with specimens obtained in the setting of no neoadjuvant therapy, the macroscopic assessment and microscopic analysis of specimens after neoadjuvant therapy is more intricate and demanding.

Overall, the pathologic processing of breast specimens is complex and challenging. This is compounded by the multidisciplinary nature of breast oncology. The management of breast cancer is dependent on the clinicopathologic results, and this information often resides in a variety of locations (e.g., the electronic medical records of the surgeon, medical oncologist, and pathologists) as well as in numerous radiology reports (ultrasounds, mammograms, MRI, and post-treatment imaging). To improve the quality and efficiency of the care of breast cancer patients, a prospective quality improvement initiative was implemented in an attempt to quantify the value of proactively alerting the pathologists to the number and location of benign and malignant findings within a mastectomy specimen through the submission of a standardized breast diagram completed at the time of surgery by the surgeon, in addition to a standard pathology form. Our aim was to determine whether the standardized breast diagram, which served as a “mastectomy lesion map,” would improve communication among surgeons and pathologists, facilitate lesion localization, and reduce time to final pathology reporting.

## Methods

A prospective quality improvement study was performed at a community breast care center at Stamford Hospital, a community-academic teaching hospital in Stamford, CT. This study was reviewed and approved by the Stamford Hospital Office of Research and Institutional Review Board (reference number QR30086). As a quality improvement initiative, this study received a waiver for requiring written informed consent from the patients. Eligible participants were age 18 years or older, diagnosed with breast carcinoma, and undergoing therapeutic mastectomy. Individuals undergoing partial mastectomy or prophylactic mastectomy were excluded from analysis. Data was collected over a continuous 12-month period: 6 months prior to routine usage of the standardized mastectomy diagram, and 6 months in which the diagram was routinely utilized.

During the first 6 months, usual pathologic processing of mastectomy specimens was performed per standard department protocol and in line with previously described protocols in the literature.^13–15^ Radiographs of total mastectomy specimens were not routinely obtained by the pathology department at our institution, regardless of the presence of microcalcifications. The following general steps were performed:

1. Record date of specimen receipt.
2. Review patient’s pertinent history.
3. Determine orientation set by the surgeon (e.g., short suture—superior, long suture—lateral, skin—anterior, double stitch—deep for skin sparing mastectomies).
4. Measure and record specimen weight and dimensions.
5. Evaluate appearance (e.g., ulcerations, puckering, nipple retraction or inversion).
6. Ink specimen (e.g., anterior superior—yellow, anterior inferior—green, posterior—black).
7. Serial section the specimen from lateral to medial.
8. Identify gross lesions and document location, size, and distance from each margin.
9. Identify previously placed clips, calcifications, and any areas of suspicion based on prior measurements (e.g., lesion at 1 o’clock, 3 cm from the nipple areola complex on diagnostic mammogram).
10. Submit sections for histology of each lesion, calcification, and area of suspicion.
11. Submit representative sections of uninvolved tissue of each quadrant.
12. Histopathologic analysis with correlation back to prior radiology and pathology reports.
13. Record date of final pathology report.

**Fig. 1:**
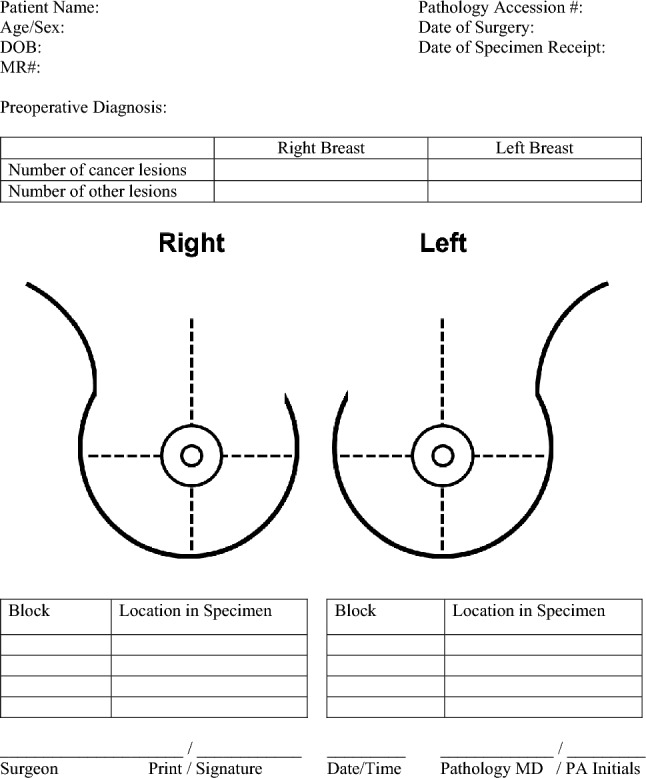
Standardized breast mastectomy diagram

In the second 6 months, a standardized mastectomy diagram was completed by the surgeons at the time of mastectomy noting the location and preoperative pathologic diagnosis of all benign and malignant lesions, and areas of radiographic concern. A copy of the standardized mastectomy diagram is shown in Fig. 1. The diagrams were usually completed during preoperative case review in preparation for surgery. Surgeon compliance was 100%. These diagrams were provided to the pathology department along with the usual standard pathology forms. The diagrams were then reviewed by the pathologists, pathology assistants, and team of technicians prior to any specimen handling, grossing, and microscopic evaluation. The processing of the specimens followed the same protocol with the following modifications:

1. The diagram was used as a mastectomy lesion map to direct lesion localization.
2. Sections submitted to histology were based on the location specified by the surgeon on the diagram.
3. All sections were labeled with a specific block code and recorded on the breast diagram form.
4. During microscopic evaluation, the pathologists correlated each histologic section with its corresponding block and location as identified on the breast diagram form.

**Fig. 2:**
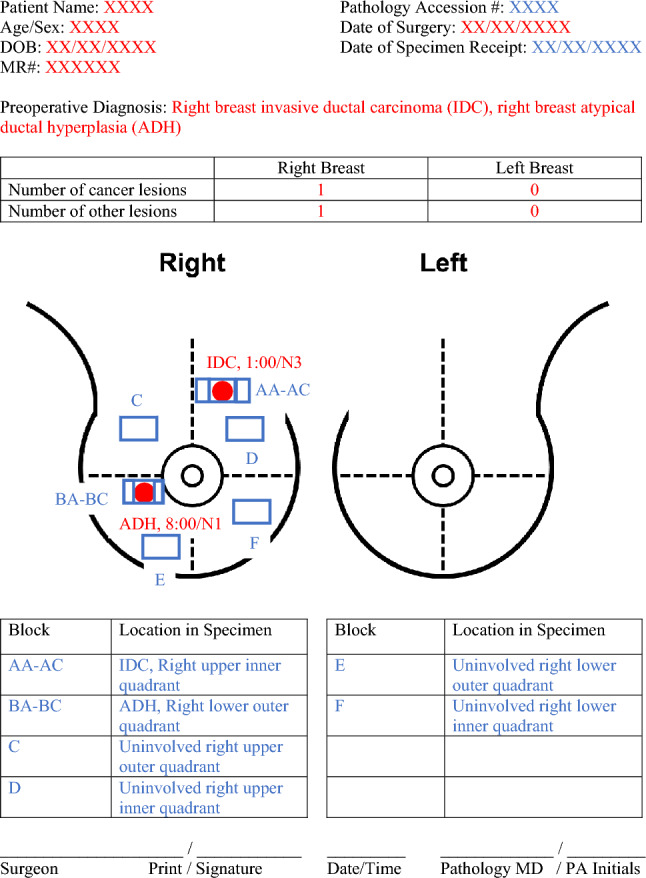
Sample of a completed mastectomy diagram. Red indicates areas annotated by the surgeon. Blue indicates areas completed by the pathology team

The entire pathology department was instructed on how to use the standardized mastectomy diagram. During specimen processing in the mastectomy diagram group, the pathologists performed the same steps in the usual processing method in addition to focusing on the lesions outlined in the mastectomy diagram to ensure that no lesions were overlooked. A sample of a completed standardized breast diagram form, first completed by the surgeon and then by the pathology team, is depicted in Fig. 2.

### Statistical Analysis

Statistical comparisons were made between the usual processing group and the standardized mastectomy diagram group. The number of expected lesions based on prior imaging and biopsies, the number of lesions identified after surgery on pathology, and the number of days between specimen receipt and the date of final pathology report was calculated. A paired samples *t*-test was performed to compare the number of expected lesions to the number of identified lesions in each group. An analysis of covariance (ANCOVA) was performed, controlling for age, sex, body mass index (BMI), race/ethnicity, and pathologic diagnosis, to compare the number of expected lesions between each group and to examine the effect of the standardized mastectomy diagram on the number of lesions identified and the time to pathology. All analyses were performed using JASP 0.14.1. The significance level was set at *p* ≤ 0.05. Values are presented as a mean ± standard deviation, unless otherwise stated.

## Results

Five surgeons participated in this study (two fellowship-trained surgical oncologists, two breast surgeons, and one general surgeon). All surgeons performing oncologic breast surgery at Stamford Hospital partook in this initiative. There was a 100% compliance in submitting the standardized mastectomy diagram and usage among the pathology team. A total of 60 patients were included in this study: 27 during the usual processing period and 33 during the period in which the standardized mastectomy diagrams were employed. Each patient provided one mastectomy specimen for analysis. Baseline characteristics were similar between the two groups (Table 1). The mean age was 59.7 ± 16.3 years in the usual processing group and 56.3 ± 13.5 years in the mastectomy diagram group. All of the patients were female and the majority identified their race/ethnicity as white.

**Table 1:** Demographic and baseline characteristics

Characteristic	Usual processing (*n* = 27)	Mastectomy diagram (*n* = 33)
Age, years^a^	59.7 ± 16.3	56.3 ± 13.5
Female, *n* (%)	26 (100%)	33 (100%)
BMI^a^	27.1 ± 5.5	25.6 ± 5.0
Race/ethnicity, *n* (%)		
Asian	2 (7.4%)	0 (0%)
Black or African American	1 (3.7%)	1 (3.0%)
Hispanic or Latino	4 (14.8%)	2 (6.1%)
Two or more races	1 (3.7%)	2 (6.1%)
White	19 (70.4%)	28 (84.8%)

^a^Values are presented as a mean ± standard deviation

A comparison of the mastectomy specimens in the usual processing and standardized diagram group is displayed in Table 2. The most common malignant pathologic diagnosis in each group was invasive ductal carcinoma. The majority of specimens were hormone receptor positive. Benign lesions were found in 74.1% of patients in the usual processing group, and 84.8% in the mastectomy diagram group. Benign lesions included findings such as fibroadenomas, atypical ductal hyperplasia, radial scars, and intraductal papillomas. Two patients in the usual processing group received neoadjuvant chemotherapy compared with three patients in the mastectomy diagram group.

**Table 2:** Comparison of mastectomy specimens implementing usual processing versus a standardized mastectomy diagram

Variable	Usual processing (*n* = 27)	Mastectomy diagram (*n* = 33)
Malignant pathology, no. of patients (%)		
Angiosarcoma	1 (3.7%)	0 (0%)
Ductal carcinoma in situ	5 (18.5%)	7 (21.2%)
Invasive ductal carcinoma	15 (55.6%)	18 (54.5%)
Invasive lobular carcinoma	1 (3.7%)	2 (6.1%)
Mixed invasive ductal and lobular carcinoma	2 (7.4%)	2 (6.1%)
No residual malignancy	3 (11.1%)	4 (12.1%)
Hormone receptor positive, no. of patients (%)	20 (74.1%)	28 (84.8%)
HER2 positive, no. of patients (%)	2 (7.4%)	6 (18.2%)
Benign pathologic lesions, no. of patients (%)	20 (74.1%)	28 (84.8%)
Neoadjuvant chemotherapy, no. of patients (%)	2 (7.4%)	3 (9.1%)
Number of lesions expected^a^	1.6 ± 0.2	1.7 ± 0.2
Number of lesions identified^a^	1.8 ± 0.2*	2.6 ± 0.2*
Time to final pathology report (days)^a^	8.3 ± 0.7*	6.1 ± 0.6*

^a^Values are presented as a mean ± standard error.

*Denotes statistically significant difference in means (*p* ≤ 0.05).

In the usual processing group, there was no significant difference in the number of lesions expected based on prior imaging and biopsies (*M* = 1.6, *SD* = 1.0) compared with the number of lesions identified (*M* = 1.8, *SD* = 1.1), *t*(26) = − 2.0, *p* = 0.06. In the standardized mastectomy diagram group, there was a significant increase from the number of lesions expected (*M* = 1.7, *SD* = 1.0) to the number of lesions identified (*M* = 2.6, *SD* = 1.4), *t*(32) = − 5.6, *p* < 0.001.

After controlling for age, sex, BMI, race/ethnicity, and pathologic diagnosis, there was no significant difference between the number of expected lesions between the usual processing group and the standardized mastectomy diagram group, with a between-group difference of 0.2 (95% confidence interval [CI] − 0.4 to 0.7; *p* = 0.6). The adjusted mean number of lesions identified significantly increased from a mean (± SE) of 1.8 ± 0.2 to 2.6 ± 0.2 after the use of the standardized diagram was initiated, for a between-group difference of 0.8 (95% CI 0.1–1.5; *p* = 0.02). In addition, the mean processing time was significantly reduced as a result of the use of the standardized diagram. Time from specimen receipt to final pathologic report decreased from 8.3 ± 0.7 days in the usual processing group to 6.1 ± 0.6 days with the use of the diagram, for a between-group difference of 2.1 days (95% CI 0.3 to 4.0; *p* = 0.02). Age, sex, BMI, race, and pathologic diagnosis did not affect time to final pathology report or the number of lesions identified.

## Discussion

The multidisciplinary nature of breast oncology necessitates effective communication among a team of surgeons, pathologists, and medical oncologists. With the advances in management and our understanding of breast carcinoma, this places a critical importance on the pathologic processing of breast specimens. The grossing of breast specimens is dependent on clinicopathologic correlation to provide accurate information for tumor staging and assessment of treatment response.^13^ Our study showed that the implementation of a standardized mastectomy diagram can improve the pathologic processing of breast specimens. Our mastectomy diagram provides the relevant patient information on one form prior to any specimen analysis and microscopic evaluation. The simplicity and ease of use made for rapid adoption. Most importantly, it enhanced communication among the surgeons and pathologists by facilitating the transfer of information. This ultimately resulted in a significant increase in the number of lesions identified on pathologic processing and a faster time from specimen receipt to final pathologic report.

Gross lesions readily identifiable on mastectomy specimens typically direct the extent of sampling during pathologic processing. However, many specimens do not have an abnormality clearly identifiable on gross examination, especially in patients with minimal disease burden. Though subjecting an entire specimen to serial sectioning techniques provides maximum detection, it is not cost-effective relative to time spent in interpretation and preparation.^16^ Systematic sampling of areas determined by informed mapping of the specimen and close correlation with radiological imaging is preferred over excessive sampling.^9^ The notion of mapping is not new, and serial slicing with subsequent imaging to create a map of the tumor bed is a well-documented method to evaluate the extent of breast cancer.^14,17^ However, our study is the first to employ a novel mastectomy lesion map prior to any pathologic processing through a standardized mastectomy diagram completed at the time of surgery. This diagram allowed the pathology team to identify areas to section with more certainty, lowering the need for over exhaustive sampling or specimen radiography. Rather than speculating where lesions and areas of suspicion were located, the diagram directed the focus of investigation. This was shown to improve the accuracy of pathologic assessment by increasing the number of lesions identified within the specimens.

Furthermore, the mastectomy diagram aided the pathologists by decreasing the burden of having to piece together information from the patient’s medical record and prior radiology and pathology reports. It also assisted with correlating the histologic slides with a lesion’s location in the specimen. Together, this allowed the pathologists to finalize each patient’s pathology report quickly and efficiently.

The faster time from specimen receipt to final pathologic report has many implications. Longer times from surgery to final pathology results can induce patient anxiety. Patients are worried about their margin and lymph node status. Most importantly, they are concerned about the impact of these pathology findings on adjuvant treatment recommendations. High levels of anxiety can impair functional status and disrupt quality of life in cancer patients.^18^ Thus, shorter times to provide important pathology findings may alleviate psychological distress and improve emotional wellbeing. Likewise, it may also strengthen patient-physician relationships by enabling timely communication.

Timeliness in pathology reports is also one measure of quality in surgical pathology.^19,20^ Timely pathology reports help prevent delays in treatment decisions and ensure that effective care is received in an appropriate timeframe. Although the reduction in pathologic processing time by 2.1 days shown in our study may not have a significant impact on overall morbidity and mortality in breast cancer patients, it represents an important improvement in quality. In low- and middle-income countries where pathology turnaround time can take weeks to months,^21^ a simple, low-cost initiative to decrease processing time may bear more significance. Therefore, initiatives to improve time from surgery to pathologic diagnosis and adjuvant treatment are warranted.

The impact of the standardized mastectomy diagram may be greater in certain subsets of patients such as those undergoing neoadjuvant treatment. Neoadjuvant chemotherapy increases the difficulty of pathologic processing of mastectomy specimens due to the need for more extensive sampling. Gross identification of residual disease is harder as the tumor bed becomes poorly defined after therapy.^9–11^ In our study, the sample size was limited to 2 patients in the usual processing group and 3 patients in the mastectomy diagram group who had neoadjuvant chemotherapy. Further studies with larger sample sizes to power an analysis investigating whether the standardized mastectomy diagram is more efficacious in improving the accuracy and timeliness of pathologic reports in patients who undergo neoadjuvant therapy are needed.

Lastly, the standardized mastectomy diagram is straightforward and easy to use, making it widely applicable across institutions. Breast surgeons are already adept at correlating areas of suspicion on two-dimensional mammographic images into three-dimensional space when localizing breast lesions during surgery. Translating a lesion’s location in three-dimensional space back onto the two-dimensional, standardized, annotated mastectomy diagram was relatively straightforward for the surgeons participating in this initiative. Likewise, the participating pathologists, who previously often referred to prior radiographic images during histopathologic analysis, found little difficulty in interpreting this visual-spatial tool. There is no reason to believe physicians at other centers would encounter difficulties implementing this initiative. Other breast cancer centers should consider adopting this simple quality improvement as it only requires copying the supplied form and having the surgeon complete the diagram with specimen submission.

## Conclusion

A standardized breast diagram completed at the time of surgery improves the quality of pathologic processing by enhancing both accuracy and efficiency. The diagram, which served as a tailored mastectomy lesion map, assisted lesion localization, reduced time to final pathology report, and aided communication among the multidisciplinary care team of breast cancer patients.
